# Predicting Violent Reoffending in Individuals Released From Prison in a Lower-Middle-Income Country: A Validation of OxRec in Tajikistan

**DOI:** 10.3389/fpsyt.2022.805141

**Published:** 2022-04-25

**Authors:** Gabrielle Beaudry, Rongqin Yu, Arash Alaei, Kamiar Alaei, Seena Fazel

**Affiliations:** ^1^Department of Psychiatry, University of Oxford, Oxford, United Kingdom; ^2^Department of Health Care Administration, California State University Long Beach, Long Beach, CA, United States; ^3^Institute for International Health and Education, Albany, NY, United States; ^4^Department of Health Science, California State University Long Beach, Long Beach, CA, United States

**Keywords:** risk assessment, external validation, clinical prediction model, OxRec, LMIC (low and middle-income countries), prison, violence, recidivism

## Abstract

**Background:**

Although around 70% of the world's prison population live in low- and middle-income countries (LMICs), risk assessment tools for criminal recidivism have been developed and validated in high-income countries (HICs). Validating such tools in LMIC settings is important for the risk management of people released from prison, development of evidence-based intervention programmes, and effective allocation of limited resources.

**Methods:**

We aimed to externally validate a scalable risk assessment tool, the Oxford Risk of Recidivism (OxRec) tool, which was developed in Sweden, using data from a cohort of people released from prisons in Tajikistan. Data were collected from interviews (for predictors) and criminal records (for some predictors and main outcomes). Individuals were first interviewed in prison and then followed up over a 1-year period for post-release violent reoffending outcomes. We assessed the predictive performance of OxRec by testing discrimination (area under the receiver operating characteristic curve; AUC) and calibration (calibration statistics and plots). In addition, we calculated sensitivity, specificity, positive predictive value (PPV) and negative predictive value (NPV) for different predetermined risk thresholds.

**Results:**

The cohort included 970 individuals released from prison. During the 12-month follow-up, 144 (15%) were reincarcerated for violent crimes. The original model performed well. The discriminative ability of OxRec Tajikistan was good (AUC = 0.70; 95% CI 0.66–0.75). The calibration plot suggested an underestimation of observed risk probabilities. However, after recalibration, model performance was improved (Brier score = 0.12; calibration in the large was 1.09). At a selected risk threshold of 15%, the tool had a sensitivity of 60%, specificity of 65%, PPV 23% and NPV 90%. In addition, OxRec was feasible to use, despite challenges to risk prediction in LMICs.

**Conclusion:**

In an external validation in a LMIC, the OxRec tool demonstrated good performance in multiple measures. OxRec could be used in Tajikistan to help prioritize interventions for people who are at high-risk of violent reoffending after incarceration and screen out others who are at lower risk of violent reoffending. The use of validated risk assessment tools in LMICs could improve risk stratification and inform the development of future interventions tailored at modifiable risk factors for recidivism, such as substance use and mental health problems.

## Introduction

The number of people in prison throughout the world is estimated at approximately 11 million, and has increased by a quarter since 2000 (1, 2). More than two thirds of the global prison population live in low- and middle-income countries (LMICs) (1). However, the majority of prison health research has been based on data from high-income countries (HICs). Conducting research in prisons is key to addressing public health challenges in these populations, such as mental health, substance misuse, infectious diseases and violence (3). However, such efforts are met with many challenges, from funding to operational constraints, (4) and evidence on people in prison in LMICs is limited (5).

Many structured risk assessment tools have been developed in criminal justice and forensic psychiatry, and there are more than 200 that estimate future violence risk (6). These tools are increasingly used in Western criminal justice to inform decisions regarding sentencing, release, probation and parole, despite significant limitations. Almost all have been developed without predetermined protocols, are not externally validated, do not report a range of recommended performance measures, and rarely include modifiable risk factors (i.e., substance misuse and mental illness) (7, 8).

In order to address these gaps, the Oxford Risk of Recidivism (OxRec) tool was developed—a novel scalable tool that includes modifiable risk factors to measure the risk of violent reoffending in people released from prison (within 1 and 2 years). Both one and two-year reoffending outcomes provide critical information to prison and probation services in making decisions about treatment, release and supervision. This tool provides an evidence-based risk assessment and is intended to complement professional judgment. It aims to facilitate assessment and linkage to care, and in turn assist in treatment allocation of individuals at high-risk of violent reoffending. OxRec is intended for use toward the end of prison sentences to prioritize those individuals for substance use and mental health community-based interventions, resources for which are often scarce. It was derived and validated using national link registries for nearly 50,000 people released from prison in Sweden (8). The model has been validated in the Netherlands, using a national sample of all individuals released from prison, reporting adequate predictive accuracy (9).

To date, risk assessment tools in criminal justice have been developed using data from HICs. In addition, external validation is often overlooked, even in HICs (10). Thus, it is not known whether these tools are applicable to LMICs, given potential differences in effects of risk factors and base rates of reoffending in various countries (11).

We report an external validation of the OxRec tool using data from a prospective cohort study in Tajikistan. Tajikistan is the poorest country in Central Asia, and one of the few lower-middle-income countries in this region, with nearly 27% of the population living in poverty (12). Such a validation could provide an approach to improve risk stratification and guide resource allocation in this LMIC setting.

## Methods

### Ethical Approval

Ethical approval for this validation study was obtained from the Institutional Review Board (IRB) of the Tajikistan Prison Organization on August 8, 2019. The study protocol is available online (see Supplementary Material).

### OxRec

OxRec is a structured violence risk assessment tool based on 14 routinely collected predictors. These risk factors can be grouped in three domains: criminal history (length of incarceration, violent index offense, previous violent crime), sociodemographic (sex, age, immigrant status, civil status, education, employment, disposable income, neighborhood deprivation) and clinical information (alcohol or drug use disorder, any mental disorder, any severe mental disorder). The contribution of each predictor to the model has been published (8). OxRec can be completed in less than 10 minutes using a freely accessible online calculator: https://oxrisk.com/oxrec-9/ (which has been translated into Russian). Finally, the OxRec model demonstrated at least comparable levels of predictive performance as the most commonly used structured instruments for violence risk, (13) and possibly better performance in external validation (14).

### Study Design

This OxRec validation study was a prospective cohort study that included individuals in prison. Enrolment took place at the two main gender-specific prisons of Tajikistan, respectively located in the cities of Dushanbe (for men) and Vahdat (for women). We identified participants during two recruitment phases. The first phase took place from August 2019 to December 2019, and the second from January 2020 to March 2020. To investigate the risk of violent reoffending, we followed up the participants from the date of their release until the outcome first occurred or the end of the study (within 12 months).

### Participants

All people in prison aged 18 years and older, set to be released within the next 6 months (regardless of their index crime or the length of their sentence), were eligible for recruitment. Prison staff randomly selected eligible persons, with the aim to recruit approximately 1,000 individuals. Following this, eligible people in prison were asked whether they wanted to take part in the study. Participation was voluntary. Oral informed consent was obtained from each study participant in keeping with recommended practice.

The data were collected by trained research staff using structured interviews and criminal records based on predetermined variables. The questionnaire comprised of 44 questions grouped into three distinct categories [i.e., risk assessment tool (OxRec), General Health Questionnaire (GHQ-12), and Self-Reporting Questionnaire (SRQ-20)]. The questionnaire was first developed in English, and then translated into Tajik and Russian for the purpose of this study. The accuracy of the translation was verified by an independent bilingual expert.

The questionnaire was the basis for the structured interviews. All interviewers were experienced clinical healthcare professionals from Tajikistan's Department of Health and Prison Organization who had not previously worked in the two selected prisons. They received training and supervision throughout the data collection process from the Institute for International Health and Education (IIHE). Participants were interviewed individually in the language of their choice—their native language being either Tajik or Russian—and interviews lasted approximately an hour. Results were recorded manually on paper, and then transferred into an anonymised and protected Excel file. Each participant was given a unique identification code, allowing for questionnaire data to be linked with recidivism data at the end of the study period.

### Outcome and Predictors

We defined the predictor and outcome variables in accordance with the original OxRec study. The primary outcome was violent reoffending within 1 year of release from prison. We did not evaluate reoffending outcomes at 2 years due to lack of sufficient follow-up data. Any crime relating to interpersonal violence (e.g., sexual offenses, robbery, illegal threats and intimidation) for which the sentence resulted in imprisonment, was defined as violent. Time until reimprisonment was measured from the release date onwards. Outcome data were retrieved from criminal records that include information from police departments and correctional facilities in Tajikistan [i.e., detention centers (“sizo”) and prisons].

### Sample Size

We assumed a 10% reoffending rate for the sample size calculation in keeping with the risk cutoff used in the original derivation study (8). The effective sample size was determined by the rule of thumb of 100 events or nonevents (whichever being less frequent) required to detect substantial differences in prognostic model performance (15–17).

### Missing Data

Due to considerable differences between the Tajik and Swedish healthcare and criminal justice systems, and issues pertaining to data availability, some definitions required adaptation (see Supplementary Table 1). Moreover, two predictor variables (i.e., immigrant status and neighborhood deprivation) were omitted from OxRec as a result of being unavailable in the Tajikistan data set. Considering that these are the two weakest predictors, the impact of their omission on the estimated model performance was minimal and further reduced by imputation to deal with missingness. Thus, we averaged out these variables using mean imputation (i.e., assigning all participants the average value from the derivation sample). There were no missing data for individual participants on the remaining predictors.

### Statistical Analysis

#### External Validation

In line with previous work (18–20), we employed an incremental approach for validating models for time to event data previously detailed in a validation study of OxRec in the Netherlands (9). Baseline characteristics of the validation cohort (Tajikistan) were first compared to the derivation cohort (Sweden), using summary measures. If there were significant differences in definitions, they were adapted to the validation cohort, to improve usability, and thus future clinical utility in the intended implementation context (i.e., Tajik prisons and probation services). We then calculated the OxRec risk score for each participant (8).

#### Model Performance

Various measures were employed to assess the validated model performance in terms of accuracy, discrimination and calibration. Accuracy is a measure of overall performance which considers calibration and discrimination simultaneously. OxRec's performance in the Tajikistan prison sample was quantified using the Brier score, whereby the model predictions are compared with the actual outcome (18). In practice, the Brier score is the average mean squared difference between these values, and can range from 0 (best performance) to 1 (worst performance) (21). Discrimination refers to the model's ability to distinguish individuals with the outcome of interest (i.e., violent offense after release from prison) from those without this outcome (18). To evaluate the model's discrimination, the area under the receiver operating characteristic curve (AUC), or *c* index, was used (22). The AUC takes values between 0.5 and 1, and represents the probability that individuals who commit violent crimes will be given a higher-risk score than those who do not reoffend (7). We also calculated sensitivity, specificity, positive and negative predictive values (PPV and NPV) for various prespecified risk thresholds to inform potential benefits (and harms) for intended management purposes. Conversely, calibration indicates the extent to which predicted and observed outcomes are in agreement. Calibration was assessed by graphical inspection of the calibration plot, and calculation of the calibration slope, and calibration-in-the-large (21). We report performance metrics for each step of the validation process for transparency purposes.

#### Model Updating

Model updating was commensurate to the predictive performance of OxRec as indicated by the above performance measures. First, we conducted simple validation. OxRec was applied to the data from Tajikistan, using the original regression coefficients and linear predictor values. In the presence of inadequate calibration, two recalibration approaches could be employed—the first being updating only the baseline risk, and the second, updating both the baseline risk and recalibrating the model coefficients via a single multiplicative recalibration value. Finally, individual parameters can be re-estimated in the event of predictor effects in the validation sample which diverge significantly from that of the derivation sample, but this final step should only be performed as a last resource (18).

Data analysis was undertaken with Stata version 17 (StataCorp LLC, College Station, TX). We reported the results and findings of this study according to the Transparent Reporting of a multivariable prediction model for Individual Prognosis or Diagnosis (TRIPOD) statement (23).

### Role of Funding Source

The funders of the study had no role in study design, data collection, data analysis, data interpretation, or writing of the report. All authors had full access to the data in the study and had final responsibility for the decision to submit for publication.

## Results

### Participants

In this prospective multicentre study, 6,853 individuals were incarcerated, 2,225 of which were eligible, in two Tajik prisons between August 2019 and March 2020. Approximately half were randomly selected (1,123 people in prison), and 1,003 provided consent for study participation. All participants had complete data on the predictor variables from the structured interview. Data on 1-year post-release outcomes was missing for 33 individuals, and thus the final validation sample included 970 people released from prison.

Compared with the Swedish derivation sample, the Tajik validation sample showed large differences in most predictors, as indicated in Table 1. The Tajik cohort included a higher proportion of female participants (13% in Tajikistan vs. 7% in Sweden), individuals with formal education (at least high school; 94 vs. 52%) and employment (64 vs. 25%). There were less unmarried Tajik individuals than Swedish ones (40 vs. 65%). Moreover, the distribution of the length of incarceration was inverted, with shorter periods of detention being less prevalent [<1% in Tajik sample vs. 69% in Swedish sample (for <6 months)], and conversely longer ones being more prevalent [85 vs. 4% (for more than 24 months)]. Whilst the Tajik sample had a higher prevalence of a violent index offense (63 vs. 38%), the proportion with previous violent crime convictions was less in the former (8 vs. 53%).

**Table 1:** Baseline characteristics of the Tajik sample compared with those of the Swedish sample.

**Variable**	**Tajik sample**	**Swedish sample**	
	**(*n* = 970)**	**(*****n*** **= 37,100)**	
**Sex**
Male	846 (87%)	93%	
Female	124 (13%)	7%	
**Age**	Median 35	Median 36	
	IQR 28 to 43	IQR 27 to 46	
**Length of incarceration**
<6 months	3 (<1%)	69%	
6–12 months	13 (1%)	16%	
12–24 months	122 (13%)	10%	
≥24 months	832 (85%)	4%	
**Violent index offense**	608 (63%)	38%	
**Previous violent crime**	76 (8%)	53%	
**Civil status**
Other	578 (60%)	35%	
Unmarried	392 (40%)	65%	
**Education**
<9 years	57 (6%)	48%	
9–11 years	784 (81%)	46%	
≥12 years	129 (13%)	6%	
**Employment**
Unemployed	345 (36%)	75%	
Employed	625 (64%)	25%	
**Income**
Low	423 (44%)	Negative (in debt)	<1%
		Zero	6%
		Low (<20th percentile)	53%
Stable	547 (56%)	Medium (20th−80th percentile)	40%
		High (>80th percentile)	1%
**Alcohol mis use**	358 (37%)	22%
**Drug mis use**	84 (9%)	23%
**Any mental disorder**	470 (48%)	22%
**Any severe mental disorder**	44 (5%)	3%

*Data are median (IQR) or n (%). Income was calculated using the international poverty line for low-income countries ($1.90 US per day)*.

The reoffending rates also differed between the validation and derivation datasets. Incidence of the primary outcome (i.e., violent reoffending over the 1-year follow-up) was similar in both cohorts [15% (or 144 people released from prison in Tajikistan) vs. 12% in Sweden]. As a result of these differences, we made adaptations to the variable definitions prior to validating OxRec to account for the prevalence of predictors (see Supplementary Table 2).

### Model Performance and Recalibration

In the unadjusted model (i.e. simple validation), OxRec had good overall discrimination, with an AUC of 0.69 (95% CI 0.64–0.73). With regard to calibration, the performance of the validation model was poor (CITL = 1.76; Slope = 0.88), indicating an underestimation of risk across all risk deciles. That is, the expected number of outcome events (with respect to the model's prediction) was lower than the observed number of violent reoffending, suggesting that the validation model required recalibration.

Thus, we recalibrated the model as per study protocol, by which we updated the baseline risk and calculated a single multiplicative recalibration value. After adjusting the baseline risk and applying the recalibration value to the model, the validation model showed good calibration (CITL = 0.11; Slope = 1.00). The new estimates for the recalibrated model (i.e. baseline risk and multiplicative recalibration shape parameter) can be found in Table 2.

**Table 2:** Recalibrated model formula.

	**Model formula**	**Baseline risk coefficient**
**Sweden**	1–S^∧^exp (Σ beta × RF)	*S* = 0.7992
**Tajikistan**	1–S^∧^exp [0.8093 × (−0.0348 ×0.3075 + 0.0259 ×0.39 −0.0098 × prison_d2 + 0.5949 × prison_d3 −0.1066 × prison_d4 Σbeta × RF)]	*S* = 0.4708

“*beta” and “RF” refer to the model coefficients and risk factors presented in the original development study Fazel et al. (8), respectively. The multiples of 0.3075 and 0.39 are adjustments to allow for the immigrant status and neighborhood deprivation variables being entirely missing in the validation study. “prison_d” corresponds to the length of incarceration, and the multiples of −0.0098, 0.5949, −0.1066 are the updated coefficients for the variable*.

Moreover, we performed a selective reestimation of coefficients for a single predictor (i.e., length of incarceration) to account for effect difference between the development and validation populations. This step was necessary as redefining the cutoffs for length of incarceration (due to considerable differences in prevalence) proved to be insufficient, which suggested that regression coefficients actually differed between the two settings (Supplementary Table 5). This differential effect could be explained by the mass amnesty announced by the Tajik government in October 2019, where most people in prison were granted an official pardon, and thus released prior to the end of their sentence.

**Figure 1:**
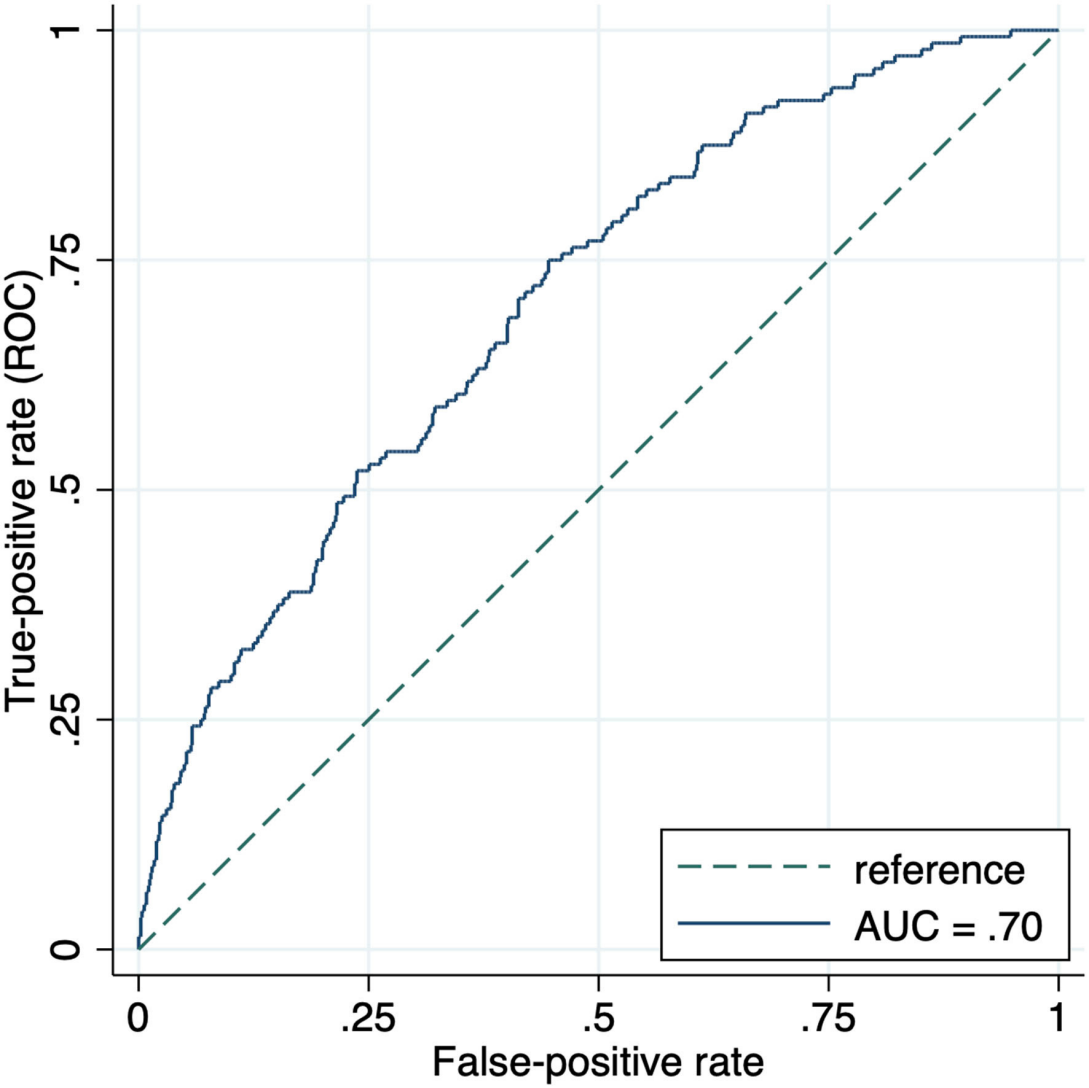
Receiver-operating characteristic curve for performance of the OxRec model in predicting violent reoffending outcome in the Tajik cohort within 1 year of release from prison. AUC, area under the receiver operating characteristic curve; ROC, receiver operating characteristic curve.

**Figure 2:**
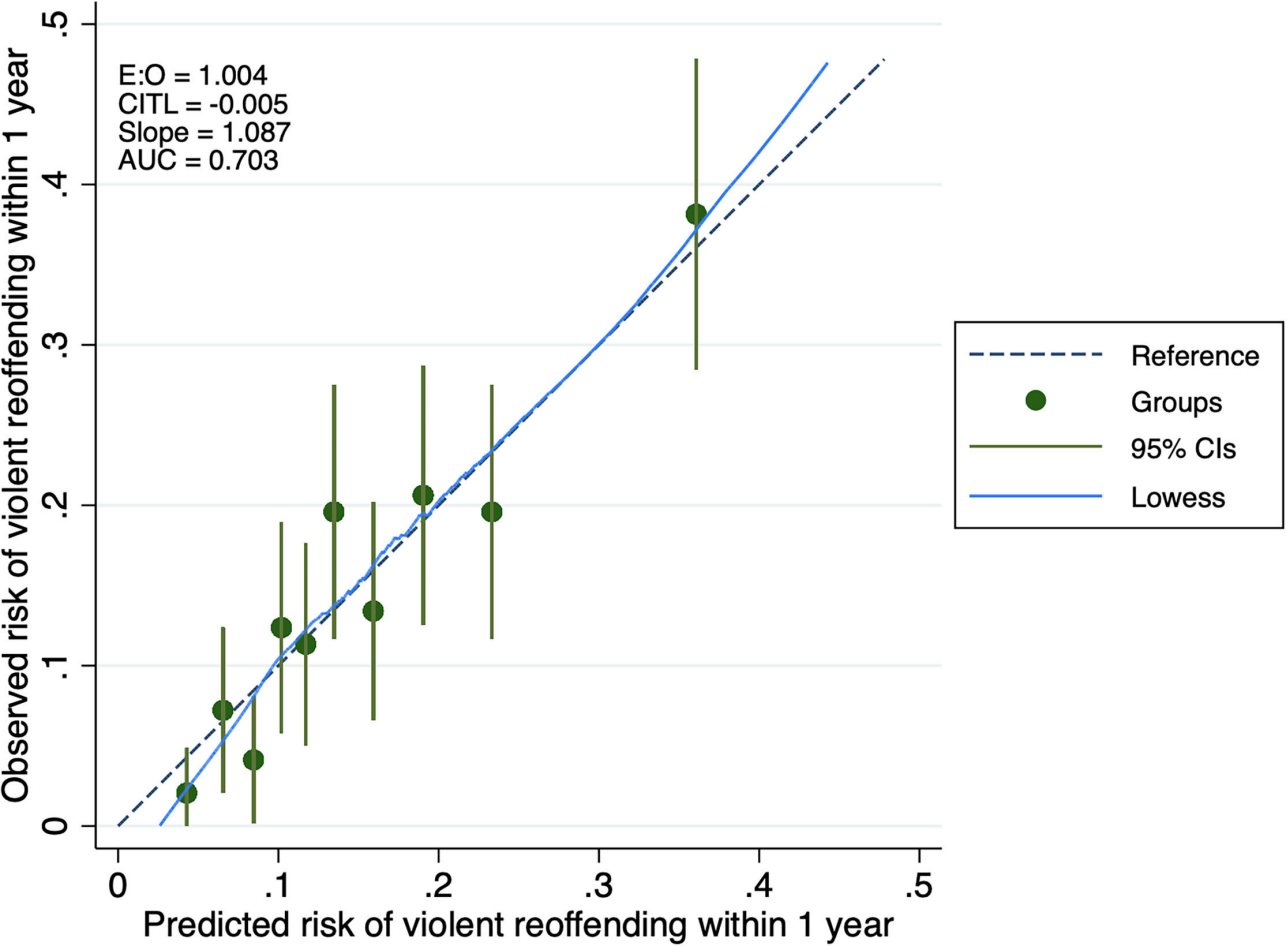
Calibration plot of the OxRec model performance in the Tajik cohort. AUC, area under the receiver operating characteristic curve; CITL, calibration in the large; E:O, ratio of expected to observed outcomes.

The revised model discrimination was indicated by an AUC of 0.70 (95% CI 0.65–0.75). Assuming a 15% risk cutoff, sensitivity was 60% (95% CI 0.52–0.68) and specificity was 65% (95% CI 0.62–0.69), whilst positive and negative predictive values were 23% (95% CI 0.19–0.28) and 90% (95% CI 0.88–0.93), respectively. This final model had a calibration slope of 1.09, and CITL was null. Calibration plots and ROC curves before and after model revision are shown in Supplementary Figure 1, and Figures 1, 2, respectively. Performance measures for the updated model are presented in Table 3.

**Table 3:** Summary of updated model performance.

	**Prevalence of reoffending**	**c-index (95% CI)**	**Risk threshold**	**Sensitivity**	**Specificity**	**PPV**	**NPV**
**Violent reoffending (1 year)**	15%	0.70 (0.66–0.75)	**5%**	99% (98–100)	8% (6–10)	16% (13–18)	99% (96–100)
			**10%**	88% (82–93)	37% (34–41)	20% (17–23)	94% (92–97)
			**15%**	60% (52–68)	65% (62–69)	23% (19–28)	90% (88–93)
			**20%**	41% (33–49)	81% (78–84)	27% (21–33)	89% (86–91)

*PPV, positive predictive value; NPV, negative predictive value*.

## Discussion

In this prospective cohort study of 970 people released from prison, we tested the performance of a risk assessment tool (OxRec), for the primary outcome of violent reoffending. 15% of participants reoffended within 1 year of release. OxRec showed good discriminative ability with an AUC of 0.70 (95% CI, 0.65–0.75). Updating the model resulted in good calibration (E:O = 1.00; CITL = 0.00; Slope = 1.09). To the best of our knowledge, this study is the first to validate a structured violence risk assessment tool for a criminal justice population in a low- or middle-income country.

### Main Findings

The observed performance of OxRec in this new setting indicated only a slight decrease in predictive performance, despite differences in social, cultural and economic contexts. In Sweden, the AUC was 0.74 for the same outcome. Country-specific variations, which tend to be particularly pronounced between HICs and LMICs, may explain differences in the distribution of participant characteristics, and in turn influence the performance of the model (24, 25).

The Tajik version of OxRec achieved similar levels of discriminative performance to other common risk assessment tools, all of which were developed in HICs. A recent systematic review of external validation studies of risk assessment tools for repeat offending (14), which included 36 studies with nearly 600,000 participants, reported AUCs that ranged from 0.57 to 0.75. Another review, which focused on tools used in US-specific correctional settings to estimate recidivism risk, found similar estimates of predictive validity (26). However, included primary studies in these prior reviews failed to report key performance measures, such as other discrimination statistics (i.e. true and false positives and negatives) and calibration, which means more detailed comparisons with OxRec are not possible. Solely using AUCs to summarize and compare the prognostic accuracy of risk tools is uninformative, as it does not allow for the consideration of false negative and false positive predictions, which vary across risk thresholds, some of which may not be clinically relevant (27, 28).

The discriminative ability of OxRec in the Tajikistan cohort was similar to that assessed in another OxRec validation study, which included both people in prison and individuals on probation from the Netherlands, as measured by the AUC [range 0.65–0.68 (for 1- and 2-year violent reoffending outcomes)]. The tool also required recalibration there, due to an overestimation of risk resulting from a lower incidence of violent reoffending [8% (Netherlands) vs. 12% (Sweden)].

The finding that the OxRec model replicated well in Tajikistan highlights the relevance of known risk factors—previously tested in a HIC context—for violent reoffending in an LMIC setting. The modifiable risk factors included in OxRec, such as mental illness and substance use disorders, are highly prevalent in people in LMIC prisons, and reflect considerable unmet mental-health needs (29). Hence, they could be directly targeted in intervention programmes for people released from prison in resource-poor settings to reduce recidivism.

Furthermore, the missingness of two predictor variables (i.e. neighborhood deprivation and immigrant status) did not materially alter the model's predictive performance. This finding is unsurprising considering that they are the weakest predictors (according to OxRec's risk factor weighting) (8). Immigrant status was found to be protective for recidivism in the Swedish studies, and therefore can be justified on ethical reasons as it might mitigate possible professional biases. However, the effect is likely to be different in LMICs.

The incidence of violent reoffending was similar across the Tajik and Swedish samples (15% in Tajikistan vs. 12% in Sweden within 1 year). Non-violent crimes are less likely to be prosecuted in LMICs such as Tajikistan, and thus that those convicted are less likely to be imprisoned, which underscores our decision to focus on violent recidivism.

### Implications

We have validated a scalable, feasible tool to predict future repeat violent crime in people released from prison in a LMIC setting. With minor adjustments, including a straightforward recalibration, the OxRec tool can provide an effective way of identifying individuals at highest risk of violent reoffending, and in turn prioritize them for community interventions upon release from prison, such as substance use clinics. Such tools are needed, particularly in LMICs where resources are limited due to low levels of investment in prison and community public health services (3). Even when available, considerable barriers to accessing resources exist for justice-involved individuals in LMICs, partly due to fear of stigmatization, lack of treatment in community settings and limited linkage between services (30).

Moreover, the performance of OxRec in terms of obstacles to external validation, such as outcome incidence, case-mix (i.e., population characteristics) and predictor effects, highlights its potential generalisability to other LMIC settings. Future research could investigate novel risk factors that might be unique to LMICs, although this may involve the development of new models (31).

As there is a paucity of research on violence risk assessment in LMICs, this study provides one approach. We highlight several challenges to risk prediction in LMICs, encountered in the process of testing the predictive ability of OxRec in Tajikistan. These should be considered prior to undertaking subsequent validations. Most assessment methods employed in HICs necessitate the involvement of trained research professionals. However, such expertise is scarce and typically too costly for LMIC settings (31). Prison health-care services in LMICs are under-resourced. This means that there is a lack of resources for conducting research, and thus data collection, especially when this involves a large number of predictors, given the absence of electronic records, or even readily available registries (32). Some data are partially missing, or reported inconsistently, whilst others are simply not available in LMICs (33). Therefore, substantial model adaptations and refinement may be required during the validation process.

### Strengths and Limitations

Our study has several strengths. We present key performance measures of discrimination (i.e., true and false positives and negatives) for various risk thresholds, in addition to the AUC. Those are often overlooked in validation studies, despite being important in terms of potential consequences for justice-involved individuals and the wider society, as they are likely to inform decisions relating to rehabilitation and public safety (7). The study design is another strength. Prospective cohort studies, as opposed to retrospective ones, offer the advantage of compiling more reliable information regarding exposures, confounders, and end points, and allow for the estimation of outcome incidence (34).

Some limitations should be noted. Several adaptations were made to variable definitions, and two predictors, neighborhood deprivation and immigrant status, were omitted. We relied on self-reported information for variables relating to mental illness and substance use disorders, which may introduce report bias especially in LMICs due to stigma (30, 35), whereas diagnosed conditions were used in the development study. However, it was not possible to rely on diagnoses in Tajikistan, and likely in other LMICs, due to the relative lack of public healthcare services. Therefore, we determined cut off scores for self-reported predictors bearing in mind prevalences in the derivation sample. We could not stratify results by sex (as assigned at birth) as the number of women included in our sample was insufficient for a female-only validation study.

## Conclusion

Many risk prediction models are available to predict repeat offending and violent crime in justice-involved individuals, which have been developed and validated in HICs. We provide a validation of a scalable risk assessment tool which could be used in Tajikistan and potentially other LMICs, to improve the consistency, transparency and accuracy of risk assessment, and linkage to community services. The use of validated tools in LMICs could improve health equity by anchoring resource allocation in an evidence-based way.

## Data Availability Statement

The dataset generated for this study and the analysis code can be provided by the authors upon reasonable request.

## Ethics Statement

The studies involving human participants were reviewed and approved by Institutional Review Board (IRB) of the Tajikistan Prison Organization. Written informed consent for participation was not required for this study in accordance with the national legislation and the institutional requirements.

## Author Contributions

SF and RY conceived and designed the study. AA and KA oversaw the data collection, data extraction, and ethical approval process in Tajikistan. GB conducted the analyses under the supervision of RY. GB also wrote the initial draft with detailed input from RY and SF. All authors contributed to the article, its revision and approved the submitted version.

## Funding

GB is funded by the Fonds de Recherche du Québec—Santé (FRQS), Grant Number 282526. SF is funded by the Wellcome Trust, Grant Number 202836/Z/16/Z.

## Conflict of Interest

SF was part of the study team that first developed OxRec. He has not received any compensation in relation to its development, use or translation. The remaining authors declare that the research was conducted in the absence of any commercial or financial relationships that could be construed as a potential conflict of interest.

## Publisher's Note

All claims expressed in this article are solely those of the authors and do not necessarily represent those of their affiliated organizations, or those of the publisher, the editors and the reviewers. Any product that may be evaluated in this article, or claim that may be made by its manufacturer, is not guaranteed or endorsed by the publisher.
