# Supplementary material for: Predicting Violent Reoffending in Individuals Released From Prison in a Lower-Middle-Income Country: A Validation of OxRec in Tajikistan
Source: Front Psychiatry. 2022 Apr 25;13:805141. doi: 10.3389/fpsyt.2022.805141 (PMC9082534; doi:10.3389/fpsyt.2022.805141)
Supplement: Supplementary file 1 [file Data_Sheet_1.docx]

**Supplementary Text 1.** Study protocol

Clinical prediction rules for risk of violent reoffending in released prisoners in Tajikistan

This project aims to examine the predictive validity of OxRec in Tajikistan. More specifically, we will test how well the OxRec can predict violent and any crime committed within 1 year (and if possible 2 years) of release from Tajikistan prisons. We will use routinely collected data including crime, mental disorders, and demographic factors such as gender, age, and employment. See appendix for a complete list of variables.

Researchers in Tajikistan will follow up a group of released prisoners for 1 year (and if possible 2 years). They will collect data on the following predictor variables: Sex, age, length of incarceration, violent index offence, previous violent crime, civil status, educational level, employment before incarceration, disposable income, a diagnosis of alcohol use disorder or equivalent, a diagnosis of drug use disorder or equivalent, a diagnosis of any severe mental illness (schizophrenia-spectrum disorder or bipolar disorder), a diagnosis of any mental disorder (or equivalent using valid proxies for mental disorder). In addition, they will collect data on the outcome variables: Any criminal/violent reoffending within 1 year of follow up and if possible within 2 years. Colleagues at the Institute for International Health and Education (IIHE) are working in Tajikistan and they will facilitate the data collection process and one part time local coordinator and three local project assistants will be hired to collect the data from three prisons and jail.

After the data are collected, researchers at the University of Oxford will perform statistical analyses, following similar procedure as the Dutch OxRec validation study (Fazel et al., 2019), and write up research reports. The public benefits may include improved violence risk assessment in prisoners and targeting of appropriate interventions, by identifying individuals who are at the highest risk of re-offending and most in need of interventions, particularly for drug and alcohol use disorders, to reduce future criminality. More specifically, if the tool is validated, the risk calculator can be put online and used in Tajikistan by criminal justice professionals and healthcare staff. For instance, the tool could be used by prison health care to help guide treatment of prisoners before their release and community linkage, especially for those who need additional substance misuse treatment on release. It can also assist case workers in planning sentencing and release arrangement. In addition, as our tool does not require any training, any health-care or criminal justice professional such as judge, probation officers, psychiatrists, nurses could use them to assist in decisions on the timing of parole and conditions associated with it. Findings will be published in peer reviewed scientific journals, and presented at relevant academic conference.

Reference: Fazel, S, Wolf, S, Vasquez Martez, M and Fanshawe, T (2019) ‘Prediction of violent reoffending in prisoners and individuals on probation: a Dutch validation study (OxRec)’ *Scientific Reports* doi: 10.1038/s41598-018-37539-x.

Appendix: List of risk factor variables

| Variable | Notes |
| --- | --- |
| Sex | Male / Female |
| Age | Calendar age. Age at release from prison. |
| Immigrant | First or second generation immigrants (born outside of Tajikistan) |
| Length of incarceration | Duration of incarceration for most recent offence. |
| Violent index crime | Most recent offence was homicide, assault, robbery, arson, any sexual offense (rape, sexual coercion, child molestation, indecent exposure, or sexual harassment), illegal threats, or intimidation. |
| Previous violent crime (before index offence) | Violent offence previous to most recent offence. |
| Civil status | At imprisonment. Other includes married, cohabiting, divorced, and widowed. |
| Highest education | Years of formal schooling (primary school onwards). |
| Employment | Employed vs. unemployed at time of incarceration. |
| Disposable income | Negative; Zero; Low (<20th percentile); Medium (20th – 80th percentile); High (>= 80th percentile). Unknown. |
| Alcohol use disorder | Previous diagnosis of alcohol use disorder (before or during incarceration). |
| Drug use disorder | Previous diagnosis of drug use disorder (before or during incarceration). |
| Any mental disorder | Previous diagnosis of any psychiatric disorder (before or during incarceration; excluding drug and alcohol use disorder). |
| Any severe mental disorder | Previous diagnosis of schizophrenia-spectrum or bipolar disorder (before or during incarceration). |

**Supplementary Table 1.** Variable definitions

| Variable | Sweden | Tajikistan |
| --- | --- | --- |
| Sex | Assigned at birth | Same definition |
| Age | Age at release from prison | Same definition |
| Immigrant status | First or second generation immigrants (self or either parent born outside of Sweden) | Not included |
| Length of incarceration | Duration of incarceration for most recent offence | Same definition |
| Violent index offence | Most recent offence was homicide, assault, robbery, arson, any sexual offence (rape, sexual coercion, child molestation, indecent exposure, or sexual harassment), illegal threats, or intimidation | Same definition |
| Previous violent crime | Any conviction for a violent offence previous to most recent offence (i.e. before index offence) | Any incarceration for a violent offence as defined above, previous to most recent offence. |
| Civil status | Unmarried vs other (At imprisonment. Other includes married, cohabiting, divorced, and widowed) | Unmarried vs other (At imprisonment. Other includes married, married more than once, and divorced). |
| Education | Lower secondary, upper secondary, post-secondary | None / high school (uncompleted), high school (completed), university (uncompleted), university (both uncompleted and completed). |
| Employment | Employed at incarceration. (Worked for at least 4 hours [based on their income information] during November before incarceration) | Employed at incarceration (including Tajik labour migrants). |
| Income | Negative/Zero/Low/Medium/High (Low: <20thpercentile, Medium: 20-80thpercentile, High: >80thpercentile). ‘Low’ and ‘Medium’ disposable income had accounted for 93%. | Low (below or equal to the international poverty line for low-income countries [1.90$ per person per day]) vs stable (above this line) |
| Neighbourhood deprivation | Principal components analysis of: mean disposable income, % welfare recipients, % unemployed, % divorced individuals, % with only primary school qualifications, % of immigrants (defined as individuals who were not born in Sweden), residential mobility rate, and crime rate. | Not included |
| Alcohol use | Diagnosis of alcohol use disorder (lifetime: before or during incarceration – ICD-8: 291, 303; ICD-9: 291, 303, 305A; ICD-10: F10). | Alcohol use (self-reported) |
| Drug use | Diagnosis of drug use disorder (lifetime: before or during incarceration – ICD-8: 304; ICD-9: 292, 304, 305 excl. 305A; ICD-10: F11-F19). | Drug use (self-reported) |
| Any mental disorder | Diagnosis of any mental disorder excluding substance use disorders (lifetime: before or during incarceration). | GHQ-12 total score ≥ 21 |
| Any severe mental disorder | ICD diagnosis of schizophrenia-spectrum or bipolar disorder (lifetime: before or during incarceration). | SRQ total score ≥ 16 |

**Supplementary Table 2.** Baseline characteristics of the Tajik sample compared with those of the Swedish sample (with adapted definitions)

| **Variable** | **Tajik sample (n = 970)** | **Swedish sample (n = 37,100)** | |
| --- | --- | --- | --- |
| **Sex** | | | |
| Male | 846 (87%) | 93% | |
| Female | 124 (13%) | 7% | |
| **Age** | Median 35 | Median 36 | |
|  | IQR 28 to 43 | IQR 27 to 46 | |
| **Length of incarceration** | | | |
| <66 months | 701 (73%) | <6 months | 69% |
| 66-72 months | 47 (5%) | 6-12 months | 16% |
| 72-84 months | 64 (7%) | 12-24 months | 10% |
| >=84 months | 158 (16%) | >=24 months | 4% |
| **Violent index offence** | 608 (63%) | 38% | |
| **Previous imprisonment** | 105 (11%) | **Previous violent crime** | 53% |
| **Civil status** | | | |
| Other | 578 (60%) | 35% | |
| Unmarried | 392 (40%) | 65% | |
| **Education** | | | |
| None / High school (uncompleted) | 57 (6%) | <9 years | 48% |
| High school (completed) | 784 (81%) | 9-11 years | 46% |
| University | 129 (13%) | ≥12 years | 6% |
| **Employment** | | | |
| Unstable | 481 (50%) | Unemployed | 75% |
| Stable | 489 (50%) | Employed | 25% |
| **Income** | | | |
| Low | 423 (44%) | Negative (in debt) | <1% |
|  |  | Zero | 6% |
|  |  | Low (<20^th^ percentile) | 53% |
| Stable | 547 (56%) | Medium (20^th^-80^th^ percentile) | 40% |
|  |  | High (>80^th^ percentile) | 1% |
| **Alcohol use** | 358 (37%) | 22% | |
| **Drug use** | 84 (9%) | 23% | |
| **Any mental disorder** | 470 (48%) | 22% | |
| **Any severe mental disorder** | 44 (5%) | 3% | |

Note. Data are median (IQR) or n (%). Income was calculated using the international poverty line for low-income countries ($1.90 US per day).

**Supplementary Table 3.** Summary of outcomes in the Tajikistan and other OxRec samples

| **Outcomes** | **Sample of people in prison *Tajikistan*** | **Comparison with Fazel et al. (2016) *Sweden*** |
| --- | --- | --- |
| 1 year violent reoffending | 15% | 12% |
| 1 year any reoffending | 15% | 44% |

**Supplementary Table 4.** Calibration performance measures for the uncalibrated OxRec tool

|  | Observed number of events | Expected number of events (uncalibrated) | Ratio, Expected: Observed  (uncalibrated) (95% CI) | Ratio of crude event rates, Sweden: Tajikistan | Ratio, Expected: Observed (recalibrated) (95% CI) | Brier score |
| --- | --- | --- | --- | --- | --- | --- |
| 1 year violent reoffending | 144 | 31 | 0.21 | 1.25 | 1.09 | 0.12 |

**Supplementary Table 5.** Risk factors included in the final recalibrated model and their hazard ratios

| **Variable** | **Multivariable adjusted hazard ratio (95% CI)** | | | |
| --- | --- | --- | --- | --- |
|  | **Tajikistan** | **Sweden** | | |
| Sex (female) | 0.29 (0.12-0.68) | 0.51 (0.45-0.57) | | |
| Age | 0.99 (0.97-1.01) | 0.84 (0.83-0.85) | | |
| Immigrant |  | 0.97 (0.92-1.02) | | |
| Length of incarceration |  |  | | |
| <66 months | 1 | <6 months | | 1 |
| 66-72 months | 1.13 (0.51-2.48) | 6-12 months | | 0.85 (0.81-0.90) |
| 72-84 months | 1.82 (1.12-3.00) | 12-24 months | | 0.69 (0.63-0.75) |
| >=84 months | 0.50 (0.29-0.85) | >=24 months | | 0.55 (0.48-0.64) |
| Violent index offence | 4.40 (2.58-7.50) | 1.53 (1.46-1.59) | | |
| Previous imprisonment | 1.89 (1.22-2.92) | Previous violent crime | | 2.41 (2.29-2.54) |
| Civil status (unmarried) | 0.85 (0.57-1.27) | 1.08 (1.02-1.15) | | |
| Highest education |  |  | | |
| None / High school (uncompleted) | 1 | <9 years | 1 | |
| High school (completed) | 1.06 (0.54-2.07) | 9-11 years | 0.83 (0.79-0.87) | |
| University | 0.81 (0.33-2.01) | ≥12 years | 0.65 (0.57-0.75) | |
| Employment | 0.77 (0.51-1.17) | 0.68 (0.63-0.72) | | |
| Disposable income |  |  | | |
| Low | 1 | Negative (in debt) | 1 | |
|  |  | Zero | 1.69 (1.11-2.57) | |
|  |  | Low (<20^th^ percentile) | 1.45 (0.96-2.19) | |
| Stable | 1.08 (0.71-1.64) | Medium (20^th^-80^th^ percentile) | 1.02 (0.84-1.24) | |
|  |  | High (>80^th^ percentile) | 1.57 (0.92-2.67) | |
| Neighbourhood deprivation |  | 1.03 (1.01-1.04) | | |
| Alcohol use disorder | 1.22 (0.85-1.75) | 1.41 (1.33-1.49) | | |
| Drug use disorder | 2.46 (1.61-3.78) | 1.51 (1.44-1.59) | | |
| Any mental disorder | 1.35 (0.96-1.88) | 1.09 (1.03-1.15) | | |
| Any severe mental disorder | 2.56 (1.13-5.78) | 1.10 (0.99-1.22) | | |

**Supplementary Figure 1.** Calibration plots and ROC curves before model revision


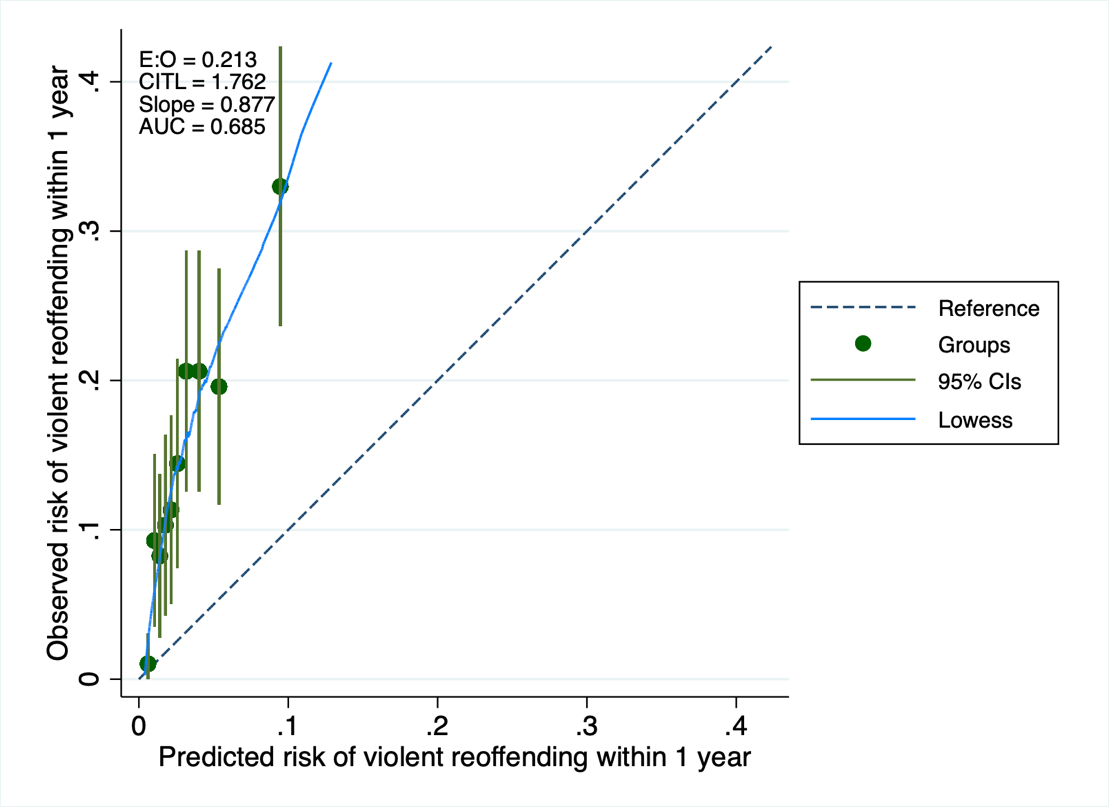

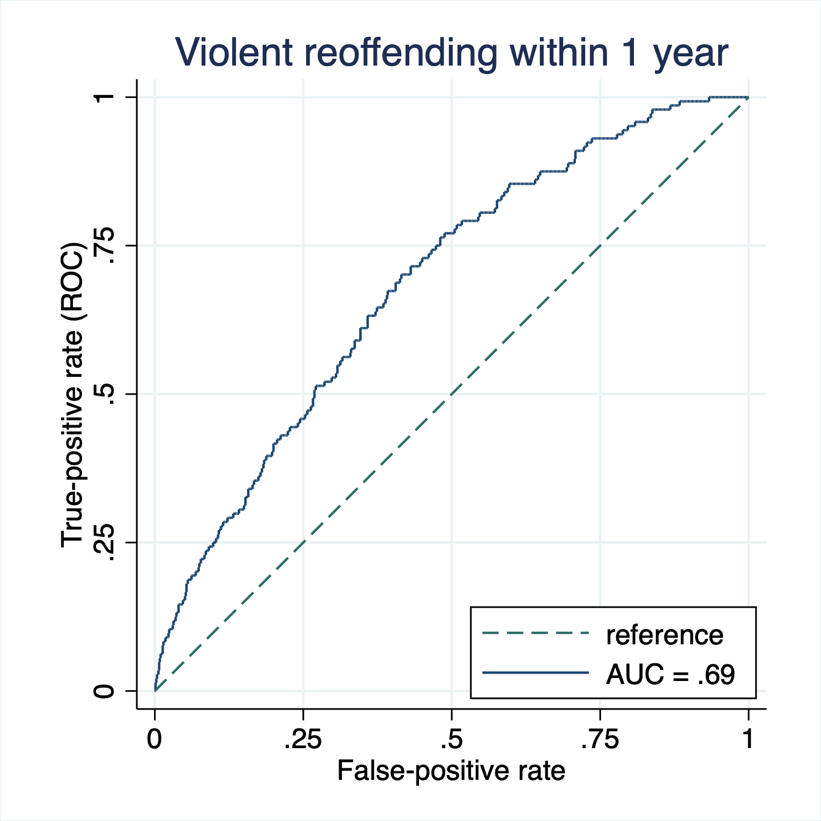


**Supplementary Figure 1a.** Calibration plot and ROC curve after simple validation.


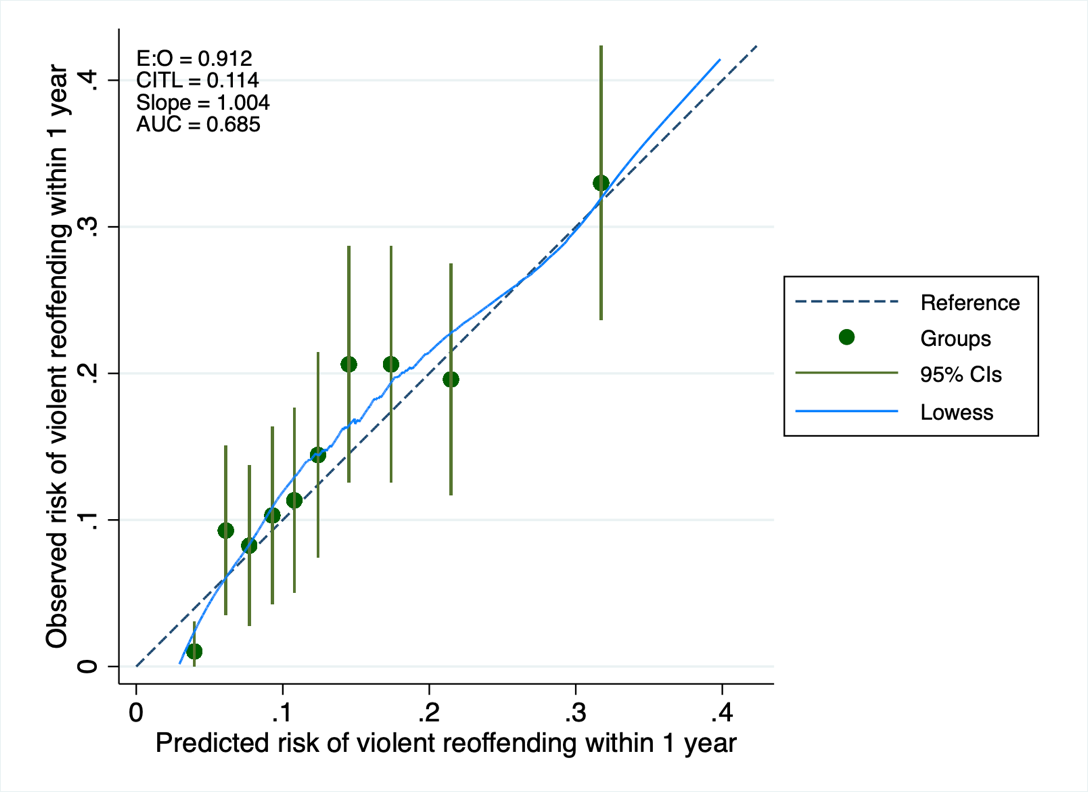

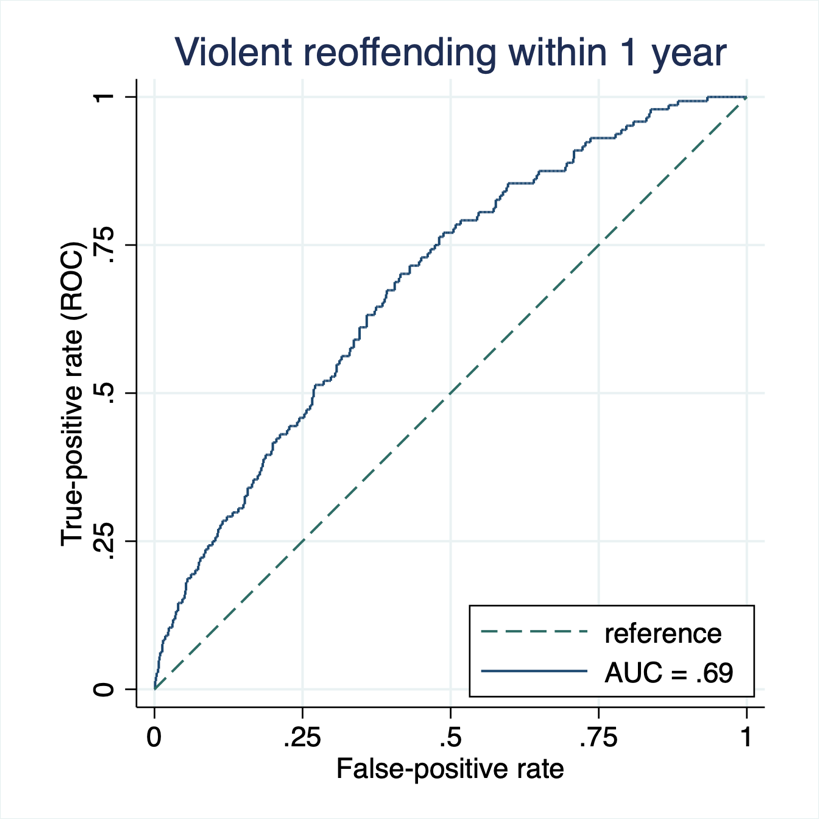


**Supplementary Figure 1b.** Calibration plot and ROC curve after recalibration.
